# Regulatory role of E3 ubiquitin ligases in multiple myeloma: from molecular mechanisms to therapeutic strategies

**DOI:** 10.3389/fcell.2025.1620097

**Published:** 2025-07-30

**Authors:** Xiaoran Chen, Jiale Zhou, Xiaoqing Dong, Yan Xu, Bing Chen

**Affiliations:** ^1^ Department of Hematology, Nanjing Drum Tower Hospital Clinical College of Nanjing University of Chinese Medicine, Nanjing, Jiangsu, China; ^2^ Department of Hematology, Nanjing Drum Tower Hospital, Affiliated Hospital of Medical School, Nanjing University, Nanjing, Jiangsu, China

**Keywords:** E3 ubiquitin ligases, multiple myeloma, pathogenesis, therapeutic targets, immunity

## Abstract

Multiple myeloma (MM) is a hematological tumor characterized by the malignant proliferation of plasma cells in bone marrow (BM). Despite the prolonged survival of MM patients, a significant amount of patients relapse or become drug resistant. This underlines the importance of the development and investigation of novel targets to improve MM therapy. Increasing evidences have shed light on the emerging roles of E3 ubiquitin ligases in MM. E3 ubiquitin ligases play an essential role in protein ubiquitination, which is involved in the regulation of protein degradation, protein-protein interactions and signal transduction. In this comprehensive review, we will summarize the current understanding of E3 ubiquitin ligases in MM and their contribution to MM therapy, which could help explore the molecular mechanisms in MM and provide potential therapeutic targets for the treatment of MM.

## 1 Introduction

**FIGURE 1:**
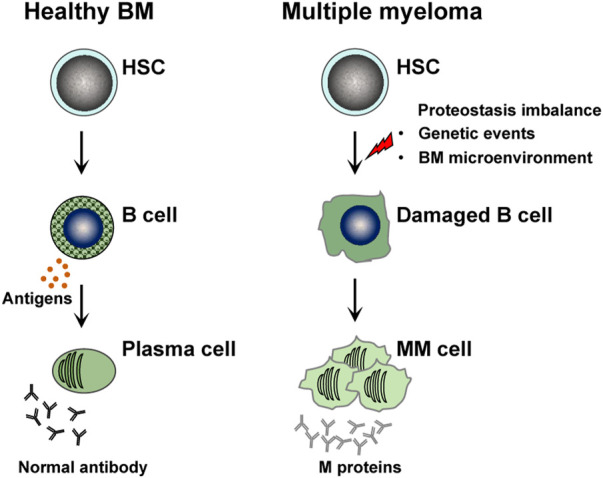
The pathogenesis of multiple myeloma.

Multiple myeloma (MM) is a hematological tumor characterized by malignant proliferation of bone marrow (BM) plasma cells and excessive production of monoclonal immunoglobulin, and often accompanied by bone pain, kidney damage, and extramedullary infiltration (Rajkumar, 2024; Malard et al., 2024) (Figure 1). As the population ages, the incidence of MM continues to rise, with a global rate of 1.92 per 100,000 in 2019, now ranking as the second most prevalent hematologic malignancy (Malard et al., 2024). In 2020, MM had a global mortality rate of 1.14 per 100,000, and the 5-year survival rate post-treatment was just 60% (Costa et al., 2017). Unfortunately, MM is still considered as an incurable disease. The application of proteasome inhibitors, immunomodulators, and monoclonal antibodies in clinical practice has significantly improved the patients survival (Garfall, , 2024), but most patients will eventually relapse and develop drug resistance (Malard et al., 2024; Hernández-Rivas et al., 2022; Gunes et al., 2024). The pathogenesis of MM is closely linked to proteostasis imbalance caused by genetic events and alterations in the BM microenvironment, involving oncogene overexpression (e.g., MYC, MAF) and mutations (e.g., KRAS, NRAS, p53) (Malard et al., 2024). However, these factors alone cannot fully elucidate the pathogenesis of MM. These clinical dilemmas prompt researchers to further clarify the underlying pathogenesis and screen more effective therapeutic targets.

**FIGURE 2:**
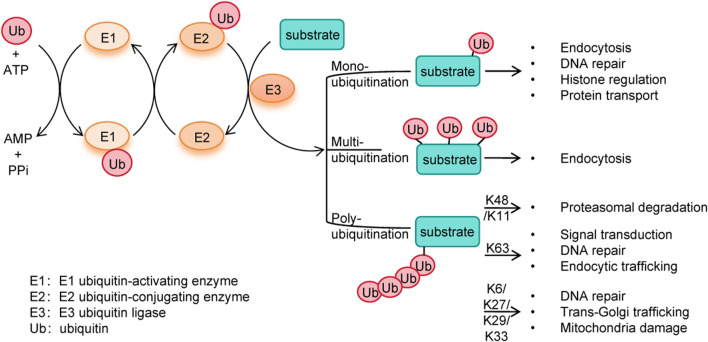
The process of ubiquitination.

In the exploration of new therapeutic targets for MM, ubiquitination has garnered significant attention due to its central role in regulating protein homeostasis (Yang and Staudt, 2015). Ubiquitination is an important post-translational modification process where ubiquitin covalently binds substrate proteins, marking them for degradation or functional/locational changes (Xu and Zhao, 2022). It regulates vital processes including protein degradation, apoptosis, DNA repair, and signal transduction. Ubiquitin (Ub) is a 76-amino-acids protein which contains seven lysine residues (K6, K11, K27, K29, K33, K48 and K63). Ubiquitination starts with ubiquitin activation by ubiquitin-activating enzymes (E1), followed by the transfer of ubiquitin to ubiquitin-conjugating enzymes (E2), and ending with the conjugation of ubiquitin to substrate protein through ubiquitin ligases (E3) (Figure 2). Ubiquitination can occur in three ways: mono-ubiquitination, multimono-ubiquitination or poly-ubiquitination (Malynn and Ma, 2010). In specific, mono-ubiquitination is involved in different cellular processes such as endocytosis, DNA repair, histone regulation and protein transport. Multimono-ubiquitination is also implicated in endocytosis. K11-linked poly-ubiquitination and K48-linked poly-ubiquitination label substrate protein for proteasomal degradation by the 26S proteasome, whereas K63-linked poly-ubiquitination is involved in signal transduction, DNA repair, endocytic trafficking and regulation of protein activity (Xu et al., 2023). Besides, K6-, K27-, K29-, K33-linked chains have been implicated in DNA repair, trans-Golgi trafficking, and mitochondria damage (Choi and Busino, 2019). Ubiquitination could interact with ubiquitin-like modifications (e.g., SUMOylation) and other post-translational modifications (e.g., phosphorylation, O-linked-N-acetylglucosaminylation (O-GlcNAcylation), acetylation, palmitoylation, lactylation). SUMOylation competes for substrate sites, modulates ubiquitin enzyme activity, and stabilizes proteins. Phosphorylation and acetylation regulate enzyme activity and signaling, while O-GlcNAcylation, palmitoylation and lactylation affect protein localization and metabolism (Zhu et al., 2025).

As important enzymes in ubiquitination, E3 ubiquitin ligases are responsible for substrate specific recognition and ubiquitination modifications. Based on structural domains, they are mainly categorized into: the RING (really interesting new gene) domain (600 members), the HECT (homology to E6AP C-terminus) domain (28 members), and the RBR (ring between ring fingers) domain (14 members). RING E3 ligases catalyze the transfer of ubiquitin from E2 to the lysine residue of the substrate, while HECT and RBR E3 ligases receive ubiquitin from E2 and transfer it to the substrate (Yang and Staudt, 2015). Numerous studies have indicated that E3 ubiquitin ligases play a pivotal role in MM pathogenesis through their ubiquitination regulation of critical proteins. For example, E3 ligase MDM2 promotes MM cell survival by mediating K48-linked ubiquitination and subsequent degradation of p53 protein (Teoh et al., 1997). From a therapeutic perspective, pharmacological targeting of ubiquitin ligases has become an important strategy in MM treatment. Among these, CRL4^CRBN^—the most representative E3 ubiquitin ligase in MM therapy—mediates the degradation of key transcription factors IKZF1/3 through its targeted drugs lenalidomide and pomalidomide, which alter CRBN’s substrate specificity, ultimately suppressing MM cell growth (Weathington and Mallampalli, 2014). Therefore, investigating E3 ubiquitin ligase targets holds significant therapeutic potential for MM treatment. Here we systematically summaries the biological functions and molecular mechanisms, therapeutic strategies, and future directions of E3 ubiquitin ligases in MM, aiming to provide new ideas and strategies for precision therapy of MM.

## 2 Ubiquitin ligases participate in the pathogenesis of multiple myeloma

**FIGURE 3:**
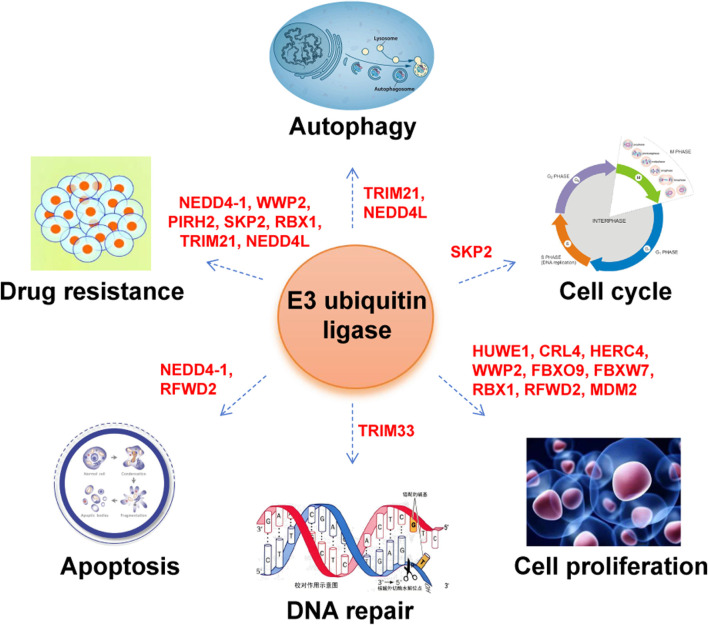
Regulatory roles of E3 ubiquitin ligases in MM.

**FIGURE 4:**
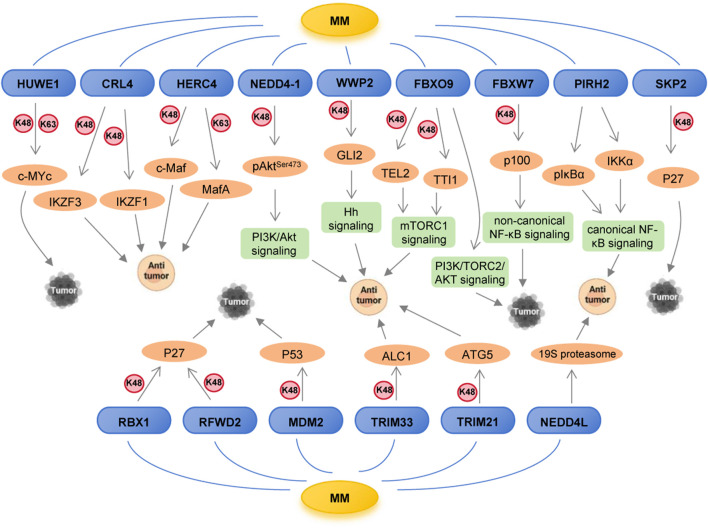
Regulatory mechanisms of E3 ubiquitin ligases in MM.

Emerging evidence has revealed that numerous ubiquitin ligases are genetically altered in MM, including mutations, amplifications, and deletions, acting as tumor suppressors or oncogenes. Supplementary Figure S1 displays the mutation types and frequencies of these E3 ubiquitin ligases in MM. Functional roles of these E3 ligases in MM mainly involved in cell proliferation, cell cycle, apoptosis, DNA repair, autophagy and drug resistance (Figure 3). From a mechanistic standpoint, these E3 ligases exert their effects through seven major ways: (1) degrading oncoproteins, such as c-Myc, c-Maf; (2) modulating tumorigenic signaling pathways, such as PI3K/AKT, NF-κB; (3) controlling cell cycle regulators, such as p27; (4) regulating apoptosis-related proteins, such as p53; (5) regulating DNA repair factors, such as ALC1; (6) governing autophagy-related proteins, such as ATG5; (7) influencing proteasome function (Figure 4; Table 1).

**TABLE 1:** Summary of E3 ubiqutin ligases implicated in MM.

E3 ligase	Mode	Target protein	Oncogenic signaling	Role	Expression	Ref
HUWE1	K48, K63	c-Myc	--	↑ proliferation	upregulated	Crawford et al. (2020)
CRL4	K48	IKZF1, IKZF3	--	↓ proliferation	--	Barankiewicz et al. (2022)
HERC4	K48	c-Maf	--	↓ proliferation	downregulated	Zhang et al. (2016)
	K63	MafA	--	↓ proliferation	downregulated	Zha et al. (2023)
NEDD4-1	K48	pAkt-Ser473	PTEN/PI3K/Akt signaling	↑ BTZ sensitivity, ↑ apoptosis	downregulated	Huang et al. (2019)
WWP2	K48	GLI2	Hh signaling	↓ proliferation, ↑ BTZ sensitivity	downregulated	Zhang et al. (2021)
FBXO9	K48	TEL2, TTI1	mTORC1 signaling	↓ proliferation	upregulated	Fernández-Sáiz et al. (2013)
	--	--	PI3K/TORC2/AKT signaling	↑ survival		
FBXW7	K48	p100	non-canonical NF-κB signaling	↑ proliferation, ↑ survival	upregulated	Busino et al. (2012)
PIRH2	--	pIκBα, IKKα	canonical NF-κB signaling	↑ BTZ sensitivity	downregulated	Yang et al. (2018)
SKP2	K48	p27	--	↓ BTZ sensitivity, ↑ cell cycle	upregulated	Malek et al. (2017), Yang et al. (2019)
RBX1	K48	p27	--	↑ proliferation, ↓ BTZ sensitivity	upregulated	Shao et al. (2022)
RFWD2	K48	p27	--	↑ proliferation, ↓ apoptosis	upregulated	Guo et al. (2021)
MDM2	K48	p53	--	↑ proliferation, ↑ survival	upregulated	Teoh et al. (1997), Maki et al. (2014)
TRIM33	K48	ALC1	--	↑ DNA repair	downregulated	McAvera et al. (2024)
TRIM21	K48	ATG5	--	↑ BTZ sensitivity, ↓ prosurvival autophagy	downregulated	Chen et al. (2023)
NEDD4L	--	19S proteasome	--	↑ BTZ sensitivity, ↑ autophagy	downregulated	Huang et al. (2022)

### 2.1 Ubiquitin ligases and oncoproteins

#### 2.1.1 HUWE1

HUWE1 is a HECT-domain E3 ubiquitin ligase, plays a pivotal role in regulating critical oncoproteins including c-Myc, p53 and MCL-1 through protein ubiquitination (Kao et al., 2018). Emerging evidence has established a strong association between HUWE1 dysregulation and tumorigenesis, with recent research identifying recurrent HUWE1 mutations in MM patients (Walker et al., 2018). Notably, HUWE1 exhibits significantly elevated expression in MM cells compared to their normal counterparts. Both *in vitro* and *in vivo* studies have demonstrated that HUWE1 is essential for sustaining MM proliferation and survival. Mechanistic investigations reveal that HUWE1 depletion induces a striking shift in ubiquitination patterns: reduction in K63-linked polyubiquitination and enhancement in K48-linked polyubiquitination. This ubiquitination switch specifically promotes c-Myc degradation, resulting in potent inhibition of MM cell growth (Crawford et al., 2020). These findings position HUWE1 as both a crucial mediator of MM pathogenesis and a promising therapeutic target. It is worth noting that potential off-target effects of HUWE1 inhibition warrant caution in clinical trial design—as hematopoietic-specific HUWE1 knockout results in bone marrow failure in murine models (King et al., 2016).

#### 2.1.2 CRL4^CRBN^

The CRL4^CRBN^ ubiquitin ligase complex represents a critical component of the cellular ubiquitin-proteasome system, composed of three core elements: RING finger domain protein (Roc1/RBX1), cullin4 (CUL4A/B) scaffold protein, and DDB1-CUL4 associated factors (substrate receptor). As the defining substrate recognition subunit of the CRL4 complex, cereblon (CRBN) plays a pivotal role in mediating the therapeutic effects of immunomodulatory drugs (IMiDs) such as lenalidomide and pomalidomide. By binding CRBN, IMiD induces conformational changes in CRBN to alter its substrate specificity. The modified complex selectively mediates ubiquitination and proteasomal degradation of transcription factors IKZF1 and IKZF3, resulting in subsequent depletion of their targets IRF4 and c-Myc. This mechanism ultimately leads to the inhibition of MM cell proliferation (Barankiewicz et al., 2022). Notably, some relapsed MM patients maintain CRBN expression and IKZF1 degradation but progress, implying potential underestimation of CRBN-independent mechanisms such as IL-6/STAT3 pathway activation (Teoh et al., 2025).

#### 2.1.3 HERC4

The basic zipper leucine transcription factors c-Maf and MafA represent critical oncogenic drivers in MM. Clinical observations reveal that overexpression of c-Maf and MafA are frequently found in MM patients, and are strongly associated with poor prognosis. These oncoproteins play multifunctional role in MM cell proliferation and survival, chemoresistance, and disease progression through transcriptional activation of critical target genes including CCND2, ITGB7, CCR1, ARK5 (Jiang et al., 2022). Contrasting with its oncogenic role in solid tumors (e.g., as a lung cancer biomarker) (Sánchez-Tena et al., 2016), HECT E3 ligase HERC4 demonstrates tumor-suppressive activity in MM through two distinct mechanisms: one way is to mediate the polyubiquitination and proteasomal degradation of c-Maf to specifically reduce c-Maf protein level (Zhang et al., 2016). While the other way is to catalyze K63-linked polyubiquitination of MafA that prevents it from phosphorylation by GSK3β, therefore inhibiting STAT3 activation and transcriptional activity (Zha et al., 2023). These coordinated actions results in the inhibition of MM cell proliferation and retardation of tumor growth. Despite high gene sequence similarity between c-Maf and MafA—both belonging to the MAF transcription factor family—HERC4 exerts differential ubiquitination modifications on these two transcription factors in this research. There is still a lack of structural biological evidence to explain this mechanistic selectivity.

### 2.2 Ubiquitin ligases and tumorigenic signaling pathways

#### 2.2.1 NEDD4-1

NEDD4-1 is a prototypical HECT-type E3 ubiquitin ligase of the NEDD4 family. In macrophages, lactate-induced lactylation of NEDD4 at K33 disrupts its binding to caspase-11, which diminishes caspase-11 ubiquitination and subsequently enhances non-classical pyroptosis, ultimately exacerbating hepatic injury (Li et al., 2024). It is widely recognized as a key oncogenic regulator in multiple malignancies (Wang et al., 2020). It is involved in the proliferation, apoptosis, cell cycle, autophagy and metastasis in many cancers. However, NEDD4-1 has been reported to recognize and ubiquitinate diverse oncogenic substrates, including N-Myc, c-Myc and RAS proteins, exerts tumor-suppressive functions in cancers such as pancreatic cancer (Zeng et al., 2014). Extensive studies have further established that NEDD4-1 can suppress tumorigenesis by regulating proliferation, apoptosis, metastasis and drug sensitivity (Zhang et al., 2020). In MM, low NEDD4-1 expression is found in patients and it is correlated with poor prognosis. Functionally, NEDD4-1 knockdown results in enhanced cell viability and bortezomib resistance in MM cells, while NEDD4-1 overexpression mediates bortezomib sensitivity through regulation of MM cell apoptosis and cell cycle. Mechanistically, NEDD4-1 mediates tumor-suppressive effects through targeting pAkt-Ser473 for ubiquitination degradation, and suppressing PTEN/PI3K/Akt pathway (Huang et al., 2019).

#### 2.2.2 WWP2

Another NEDD4 family protein, WWP2, regulates a wide range of biological processes, including DNA repair, gene expression, signal transduction, and cell-fate decisions. As such, WWP2 plays a key role in normal physiology and diseases, such as immune regulation and tumorigenesis (You et al., 2024). In MM, WWP2 is significantly downregulated in the bortezomib-resistant cells. WWP2 overexpression restricts MM tumor growth and enhances cell sensitivity to bortezomib treatment *in vitro* and *in vivo*. It has been investigated that WWP2 mediates the ubiquitination and degradation of GLI2, a core transcription factor in the activation of Hedgehog (Hh) pathway. Hence, DKK1-induced WWP2 downregulation leads to the stabilization of GLI2 protein and hyperactivation of Hh signaling, contributing to bortezomib resistance in MM cells (Zhang et al., 2021). However, WWP2-targeted therapeutic strategies present significant clinical challenges. Emerging evidence indicates that WWP2 overexpression suppresses TLR3-mediated immune response, which may facilitate tumor immune evasion (Yang et al., 2013). These immunosuppressive effects pose particularly serious concerns in the context of MM immunotherapy.

#### 2.2.3 FBXO9

SCF ubiquitin ligase complex consists of an adaptor protein SKP1, a scaffold protein cullin1 and a F-box protein (Zhou et al., 2013). The F-box protein serves as the substrate receptor that determines targets specificity for ubiquitination. FBXO9, a member of the F-box protein family, has been identified as a critical regulator in MM. Study shows that FBXO9 exhibits significant overexpression in MM cells and plays dual roles by inhibiting cell proliferation and maintaining cell survival. FBXO9 exerts its biological effects through two distinct pathways. On one hand, FBXO9 is responsible for ubiquitination-mediated degradation of TEL2 and TTI1, which leads to the downregulation of mTORC1 signaling pathway and the inhibition of MM cell growth. On the other hand, PI3K/TORC2/AKT signaling cascade is also activated by FBXO9 to sustain cell survival (Fernández-Sáiz et al., 2013). Studies demonstrate that hyperactivated mTORC1 downstream of insulin/IGF-1 signaling enhances S6K1 activity, leading to phosphorylation of insulin receptor substrate 1 (IRS1) at multiple inhibitory sites. This negative feedback loop suppresses PI3K signaling and attenuates mTORC2 activation (Wang et al., 2024). A similar regulatory mechanism may operate in MM, as suggested by prior findings.

#### 2.2.4 FBXW7

As another prototypical F-box protein, FBXW7 serves as the substrate recognition component of the SCF ubiquitin ligase complex. It plays pivotal roles in maintaining fundamental biological processes including cell cycle, apoptosis and differentiation. This critical tumor suppressor orchestrates the degradation of multiple oncoproteins such as cyclin E, c-Myc, c-JUN, NOTCH, and MCL1. It has been confirmed that FBXW7 gene mutations or downregulations have been found in many types of malignant tumors, which facilitate the uncontrolled proliferation, enhanced invasion and migration, and drug resistance of cancer cells (Qi et al., 2024). While traditionally characterized as a tumor suppressor, FBXW7 exhibits unique functionality in MM. The oncogenic mechanism shows that FBXW7 mediates K48-linked poly-ubiquitination degradation of p100 and triggers proteasomal processing to p52, thus leading the activation of the non-canonical NF-κB signaling pathway to support the growth and survival of MM cells (Busino et al., 2012). The context-dependent nature of FBXW7 function may account for the identification of its non-canonical role in MM.

#### 2.2.5 PIRH2

PIRH2 represents a recently identified ubiquitin ligase induced by p53 activation. It possesses intrinsic RING-dependent ubiquitin ligase activity for polyubiquitination and subsequent proteasomal degradation. PIRH2 functions as oncogenic role through targeting the p53 family proteins, p53, Chk2, p27Kip1, Twist1 and others, participating in such cellular processes as cell proliferation, cell cycle regulation, apoptosis and cellular migration (Daks et al., 2022). Intriguingly, PIRH2 demonstrates tumor-suppressive activity in MM, with emerging evidence establishing it as a promising therapeutic target in this malignancy. Research shows that PIRH2 overexpression enhances bortezomib sensitivity in MM cells. Mechnistic studies demonstrate that PIRH2 reduces phosphorylated IκBα (pIκBα) levels and IκB kinase α (IKKα) expression, subsequent inhibiting the canonical NF-κB signaling pathway in MM cells (Yang et al., 2018). The functional dichotomy of PIRH2 in MM may reflect tissue-specific substrate reprogramming, potentially involving the loss of its p53-binding capacity in this malignancy. While PIRH2 has been shown to downregulate both pIκBα and IKKα, the underlying molecular mechanisms remain to be elucidated, representing an important avenue for future research.

### 2.3 Ubiquitin ligases and cell cycle regulators

#### 2.3.1 SKP2

SKP2 is a critical F-box protein of the SCF ubiquitin ligase. It participates in multiple cellular functions such as cell proliferation, cell cycle, metabolism, and tumorigenesis by contributing to the ubiquitination and subsequent degradation of several specific tumor suppressors. Among them, SKP2 is responsible for the degradation of several cell cycle protein-dependent kinase (CDK) inhibitor, such as p27 (Asmamaw et al., 2020). P27 is an important cell cycle regulator that induces a cell cycle arrest and inhibits the G1/S transition through negatively regulating CDK2 and CDK4. SCF^SKP2^-mediated K48-linked poly-ubiquitination marks p27 for degradation that permits the CDK-dependent transition from a quiescent to proliferative state. SKP2 interacts with O-GlcNAc transferase and undergoes O-GlcNAcylation at Ser34, which strengthens its binding to SKP1 and prevents its degradation mediated by the anaphase-promoting complex/cyclosome (APC/C)-Cdh1. This post-translational modification enhances SKP2 stability and E3 ligase activity, promoting p21/p27 degradation and accelerating the G1-S phase transition, thereby driving hepatocellular carcinoma (HCC) cell proliferation (Feng et al., 2024). In addition, increased SKP2 expression and reduced p27 levels are frequent in human cancers and are associated with therapeutic resistance (Asmamaw et al., 2020). Particularly in MM, SKP2 is highly expressed in bortezomib-resistant patients and is a negative prognostic indicator for progression-free survival and overall survival. Knockdown of SKP2 stabilizes p27 expression and enhances bortezomib sensitivity of MM cells. Hence, the novel SCF^SKP2^ inhibitor DT204 could trigger synergistic anti-myeloma activity with bortezomib and overcame bortezomib resistance, which opens up additional possibilities for the development of novel drug combinations (Malek et al., 2017). Similarly, another SKP2 inhibitor C1 prevents SKP2-mediated ubiquitination and degradation of p27, which results in p27 accumulation and cell cycle arrest, leading to MM cell apoptosis (Yang et al., 2019). It remains unclear whether SKP2 overexpression in MM is regulated through an O-GlcNAcylation-dependent mechanism similar to that observed in HCC, warranting further exploration.

#### 2.3.2 RBX1

RBX1 serves as an indispensable component of SCF ubiquitin ligase that is vital to ubiquitin ligation. Extensive research has established RBX1’s pivotal oncogenic involvement in cancer cell proliferation, cell senescence, survival, cell cycle and apoptosis (Wei and Sun, 2010). Indeed, experimental evidence reveals that RBX1 knockdown produces potent anti-myeloma effects manifested by growth inhibition *in vitro*, tumor suppression *in vivo* models and the decreased chemoresistance. Similarly in mechanism, RBX1 enhances the ubiquitination and degradation of p27 (Shao et al., 2022).

#### 2.3.3 RFWD2

Multiple literature have reported that RFWD2 is engaged in tumorigenesis via meditating several biological processes like transcription, DNA repair, cell cycle and apoptosis. However, RFWD2 plays dualistic oncogenic/tumor-suppressive roles across different cancer types (Song et al., 2020). It demonstrates that MM patients with elevated RFWD2 expression achieve adverse clinical outcomes and drug resistance. Experimental study demonstrates that RFWD2 knockdown hinders cellular growth and triggers apoptosis in MM cells. Mechanism study reveals that RFWD2 controls MM cellular proliferation through regulating the ubiquitination and degradation of p27 via the formation of RFWD2-RCHY1 protein complex (Guo et al., 2021). While p27 degradation typically depends on T187 phosphorylation (mediated by CDK2) (Tsytlonok et al., 2019), whether RFWD2 recognizes this identical phosphodegron remains experimentally unverified.

### 2.4 MDM2 and apoptosis-related proteins

Ubiquituin ligase MDM2 is a master regulator of p53 tumor suppressor protein, which direct binding to p53 to inhibit transcriptional activity and promote ubiquitination and proteasomal degradation. Existing studies have shown that MDM2 amplification and overexpression leads to therapy resistance in different tumor entities (Brummer and Zeiser, 2024). Meanwhile, frequent MDM2 overexpression correlates with aggressive MM disease phenotypes, including enhanced survival and proliferative capacity of MM cells (Teoh et al., 1997). Therefore, MDM2 inhibition could be a target for the treatment of MM. It has now been demonstrated that MDM2 inhibitor induces cell apoptosis through activation of a p53-mediated cell death program. Besides, it also exerts synergistic effects in combination with the immunomodulator lenalidomide and overcome lenalidomide resistance (Maki et al., 2014). The synergistic mechanism likely involves MDM2 inhibition-mediated restoration of p21 levels, driven by p53-dependent transactivation of p21. This mechanism may underlie the ability of MDM2 inhibitor to overcome lenalidomide resistance. These current evidences support that MDM2 inhibitors as promising candidates for MM treatment and combination regimens with existing therapies.

### 2.5 TRIM33 and DNA repair factors

TRIM33, a member of the tripartite motif (TRIM) family, is a chromatin-associated ubiquitin ligase with diverse functional roles in hematopoiesis, embryonic development, immunity, mitosis and DNA repair (Boutanquoi et al., 2020). TRIM33 serves as an oncogenic cofactor that stabilizes fusion proteins in AML (Chang et al., 2024). As a tumor suppressor, TRIM33 has been implicated in several cancer types, such as hepatocellular carcinoma (Herquel et al., 2011). TRIM33 plays a role in PARP-dependent DNA damage response (DDR) through interaction with chromatin remodelling enzyme ALC1, facilitating ALC1 removal from DNA lesion sites, which is important for maintenance of genome stability (Kulk et al., 2013). Genetic screening studies further indicate a role for TRIM33 in the DDR, demonstrating the TRIM33 recruitment to DNA damage foci and TRIM33 deficiency leads to accumulation of γH2AX and micronuclei (Kim et al., 2019). It has been demonstrated that low TRIM33 expression is associated with poor prognosis in MM patients. Similarly in mechanism, TRIM33 loss impairs ALC1 ubiquitination, leading to accumulated DNA damage and defective double-strand break repair. These consequently accelerates genomic instability; thus sensitizing MM cells to PARP inhibitors (McAvera et al., 2024). The diametrically opposed roles of TRIM33 in AML versus MM may reflect its differential binding partner selection, with ALC1 interaction emerging as a key determinant of its genome-protective phenotype in MM.

### 2.6 TRIM21 and autophagy-related proteins

Autophagy represents an evolutionarily conserved lysosomal degradation pathway that plays critical roles in the cellular survival, homeostasis, and drug resistance in MM (Milan et al., 2016). As a RING-domain ubiquitin ligase, TRIM21 exhibits diverse functions in inflammation, autoimmunity and cancer. TRIM21 functions as tumor-promoting effects in certain malignancies and tumor-suppressive functions in others (Chen et al., 2022). It not only interacts with multiple autophagy regulators but also participates in drug resistance in various cancers (Alomari, 2021). Specifically in MM, low TRIM21 expression is a factor for relapse and contributes to bortezomib resistant, while TRIM21 overexpression enhances bortezomib sensitivity. Furthermore, TRIM21 inhibits prosurvival autophagy via K48-linked poly-ubiquitination of ATG5 for proteasomal degradation to enhance bortezomib-induced cell death (Chen et al., 2023). Such investigations remind us a combination of autophagy inhibitors may be an option to enhance the efficacy of bortezomib in treating MM patients with low TRIM21 expression.

### 2.7 NEDD4L and proteasome function

The 19S proteasome is the regulatory subunit of the 26S proteasome that degrades ubiquitinated proteins and is responsible for bortezomib resistance (Barrio et al., 2019). Autophagy demonstrates paradoxical roles in cancer biology depending on the cancer type and disease stage. On one side, it functions as a pro-survial mechanism through enhancing cellular stress tolerance. On the other side, it acts in a tumo-suppressive pathway through eliminating damaged organelles and proteins (Zhang and Shi, 2023). Another member of the NEDD4 family of HECT-type ubiquitin ligases, NEDD4L is involved in the autophagy regulation and mediation of drug sensitivity in diverse cancers (Zhang and Shi, 2023). It exerts a tumor suppressive role evidenced by poor prognosis in cancer patients with low NEDD4L expression, such as MM. Overexpression of NEDD4L increases bortezomib sensitivity in MM cells through binding the 19S proteasome, limiting its proteolytic function and enhancing autophagy (Huang et al., 2022). While NEDD4L-mediated inhibition of 19S proteasome activity would theoretically lead to ubiquitinated protein accumulation, this phenomenon was not experimentally verified in this study. Moreover, the potential induction of bortezomib-like peripheral neuropathy (Yan et al., 2021) by proteasome inhibition through NEDD4L activators - a well-documented toxicity - was completely overlooked in current animal models. These findings suggest both the application of NEDD4L as a predictive biomarker for MM patients’ bortezomib response, as well as the development of NEDD4L activators in combination with bortezomib and autophagy regulators as a novel therapeutic approach for MM.

## 3 Targeting ubiquitin ligases in the treatment of multiple myeloma

**FIGURE 5:**
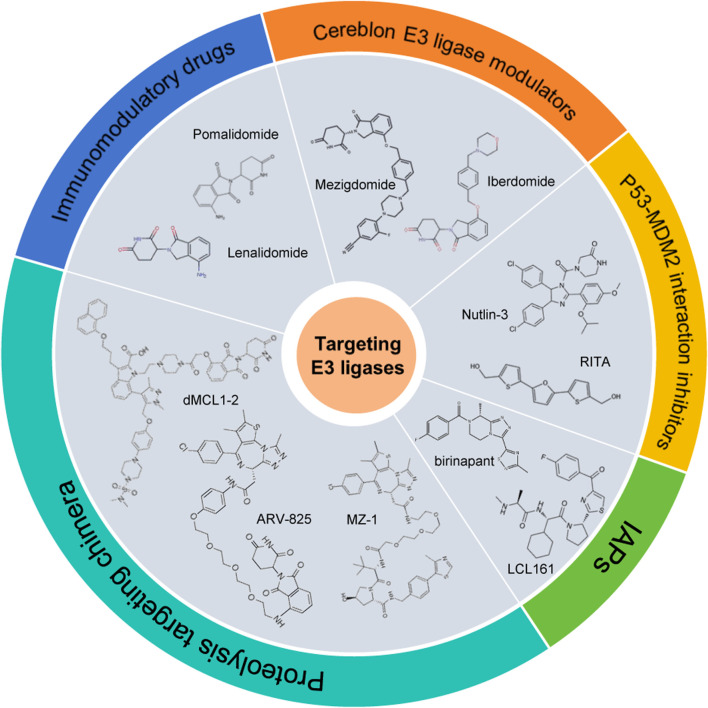
Pharmacological agents targeting the E3 ubiquitin ligases.

A deeper understanding of the biological functions and molecular mechanisms of ubiquitin ligases in MM not only helps to elucidate the pathogenesis of MM, but also provides a theoretical basis for the development of novel targeted therapy strategies. An increasing number of ubiquitin ligases have been investigated as novel therapeutic targets for the treatment of MM, opening up new avenues to overcome the limitations of existing treatments. Listed below are the ubiquitin ligase-targeting therapeutics (Figure 5; Table 2).

**TABLE 2:** Therapeutic strategies targeting E3 ubiquitin ligases in MM.

Therapeutic strategy	Representative drug	Targeted E3 ligase	Research phase	Advantage	Challenge
IMiDs	Lenalidomide	CRL4^CRBN^	Clinically applied	Initial/recurrent MM	Drug resistance (CRBN dependent mechanism/proteasome dysregulation/signal pathway compensation/microenvironment adaptation)
	Pomalidomide	CRL4^CRBN^	Clinically applied	Lenalidomide-resistant MM/relapsed/refractory MM	
CELMoDs	Mezigdomide	CRL4^CRBN^	Phase Ⅲ clinical	RRMM/acquired resistance to IMiDs	
	Iberdomide	CRL4^CRBN^	Phase Ⅲ clinical	RRMM/IMiDs-refractory disease	
PROTACs	ARV-825	CRL4^CRBN^	Early clinical	Potent BRD4 degradation capacity/significant anti-proliferative effects	Excessive molecular weight/off-target effects
	MZ-1	VHL	Preclinical/early clinical	BRD4-targeting PROTAC/synergistic effects with the trametinib	Excessive molecular weight/unfavorable pharmacokinetic
	dMCL1-2	CRL4^CRBN^	Preclinical	Potent MCL1 degradation capacity/reduce on-target toxicity	Excessive molecular weight
P53-MDM2 interaction inhibitors	Nutlin	MDM2	Early clinical	WT-p53 MM/synergistic activity with BTZ	No effect on patients with p53 mutations
	RITA	MDM2	Preclinical	MDM2 inhibitor-resistant MM/synergistic effects with Nutlin-3	Inadequate efficacy/narrow therapeutic indices
Inhibitors of apoptosis proteins	LCL161	IAPs	Phase II clinical	Induce direct apoptosis/combination therapy with JAK2-specific inhibitor	Inadequate efficacy/narrow therapeutic indices
	birinapant	IAPs	Preclinical	RRMM/Synergistic activity with BTZ/activate apoptotic and inactivate non-canonical NF-κB	Inadequate efficacy/TNF-α- related toxicity

### 3.1 Immunomodulatory drugs

#### 3.1.1 Lenalidomide

Lenalidomide is a second-generation immunomodulatory drug (IMiD) that has revolutionized MM treatment. By binding to CRBN, lenalidomide recruits CRL4^CRBN^ E3 ubiquitin ligase to induce the proteasomal-dependent degradation of transcriptional factors IZKF1 and IKZF3. Degradation of IKZF1/3 downregulates the critical oncoproteins IRF4 and c-MYC, resulting in direct anti-myeloma activity with suppressed proliferation and survival of MM cells (Krönke et al., 2014). Besides, lenalidomide exhibits immunomodulatory effects through enhancing T-cell and NK-cell activity, increasing IL-2 and IFN-γ production, and reducing regulatory T-cell function (Colley et al., 2024). It shows that CRBN expression is required for the anti-myeloma activity of IMiDs. However, during the course of lenalidomide treatment, almost all patients relapse due to the acquisition of drug resistance, which is caused by deregulated CRBN expression, proteasome dysregulation, signal pathway compensation or microenvironment adaptation.

Besides, derivatives of lenalidomide are also being actively explored. Recent studies have demonstrated that modifying the six-position substituent of lenalidomide can enhance substrate selectivity in anti-hematological carcinogenesis while eliminating teratogenic effects associated with certain derivatives. Notably, 6-fluoro-lenalidomide exhibits superior IKZF1 and IKZF3 degradation potency compared to lenalidomide, along with pronounced anti-proliferative activity in MM cell lines. While 6-chlorolenalidomide shows reduced anti-proliferative effects relative to both lenalidomide and its 6-fluoro counterpart, it presents a significant advantage by avoiding degradation of SALL4 and PLZF proteins - the putative mediators of thalidomide’s teratogenicity. These findings establish a promising framework for developing novel thalidomide derivatives with optimized therapeutic efficacy and reduced toxicity profiles for MM treatment (Yamanaka et al., 2023).

#### 3.1.2 Pomalidomide

Pomalidomide, another second-generation IMiD, is approved for relapsed/refractory multiple myeloma (RRMM). Structurally and mechanistically similar to lenalidomide, pomalidomide exerts direct tumoricidal effects by binding CRL4^CRBN^ E3 ubiquitin ligase, leading to the degradation of IKZF1/IKZF3, while also enhancing T-cell and NK-cell activation for immunomodulation. However, pomalidomide exhibits distinct anti-proliferative and pro-apoptotic mechanisms compared to lenalidomide. Specifically, it induces caspase-8-dependent apoptosis and upregulates p21^WAF1^ (independent of p53), triggering G1/G0 cell cycle arrest and suppressing tumor growth. Within the BM microenvironment, pomalidomide potently inhibits TNF-α, reducing the secretion of growth-promoting cytokines (IL-6, bFGF, VEGF) and promoting MM cell death (Richardson et al., 2013). A key differentiator is pomalidomide’s unique ability to degrade ARID2 (a PBAF complex subunit), which suppresses MYC expression and overcomes lenalidomide resistance (Yamamoto et al., 2020). Given that heavily pretreated MM patients often exhibit immune dysfunction, pomalidomide’s potent anti-proliferative activity makes it an optimal therapeutic choice in this setting. Clinically, the pomalidomide-dexamethasone combination demonstrates strong synergistic tumoricidal effects, offering a promising treatment strategy for RRMM patients (Siegel et al., 2020). The synergistic mechanism likely involves pomalidomide-induced IKZF3 degradation, leading to IRF4 and MYC downregulation, which collectively results in strong, synergistic effects on the induction of apoptosis (Rychak et al., 2016).

### 3.2 Cereblon E3 ligase modulators

#### 3.2.1 Mezigdomide

The emergence of resistance to traditional IMiDs has driven the development of cereblon E3 ligase modulators (CELMoDs), a novel class of agents characterized by enhanced substrate degradation efficiency and expanded substrate spectrum. Mezigdomide is a novel CELMoD reagent that induces polyubiquitination and degradation of the IKZF1 and IKZF3 more rapidly and effectively than lenalidomide and pomalidomide, resulting in the downregulation of IRF4 and c-MYC expression. Meanwhile, Mezigdomide also significantly promotes the production of IL-2 and IFN-γ, enhancing the activation of T cells and anti-tumor immunity (Patel et al., 2024). Preclinically, mezigdomide exhibits significantly stronger anti-proliferative and tumoricidal effects than lenalidomide or pomalidomide, even in MM cell lines with low or mutated cereblon protein or with acquired resistance to IMiDs (Hansen et al., 2020). These properties make it a promising therapeutic option for RRMM. Clinical trials are currently underway to evaluate its safety and efficacy in both newly diagnosed and RRMM.

#### 3.2.2 Iberdomide

Iberdomide represents a clinically significant advancement in the CELMoD class, demonstrating potent anti-proliferative activity against MM cell lines, including those with acquired resistance to lenalidomide and pomalidomide due to CRBN dysregulation (Bjorklund et al., 2020). It exhibits significantly greater potency in inducing cellular degradation of IKZF1 and IKZF3 compared to traditional IMiDs (Matyskiela et al., 2018). In addition to its direct anti-myeloma effects through IKZF1 and IKZF3 degradation, iberdomide exhibits robust immunomodulatory activity including activation of T cells and NK cells in the BM microenvironment of RRMM patients (Van Oekelen et al., 2024). Consistent with this, another study demonstrates that iberdomide’s clinical mechanisms of action are driven by both its cell-autonomous effects overcoming CRBN dysregulation in MM cells, and potent immune stimulation that augments anti-tumor immunity (Amatangelo et al., 2024). This comprehensive mechanism profile positions iberdomide as a promising therapeutic option for RRMM patients, particularly those with IMiDs-refractory disease. Its ability to simultaneously target myeloma cells and enhance anti-tumor immunity offers a unique therapeutic advantage in the evolving MM treatment landscape.

### 3.3 Proteolysis targeting chimera

Proteolysis-targeting chimera (PROTAC) technology represents an innovative approach for targeted protein degradation. This bi-functional molecule consists of three key components: (1) a ligand that binds to the protein of interest (POI), (2) an E3 ubiquitin ligase-recruiting ligand, and (3) a linker connecting these two moieties. By bringing the E3 ubiquitin ligase into proximity with the target protein, PROTACs facilitate ubiquitination and subsequent proteasomal degradation of the POI. Several E3 ligases with known small-molecule ligands—including CRBN, VHL, IAP, and MDM2—have been successfully utilized in PROTAC design (Li and Song, 2020).

BRD4, a member of the bromodomain and extra-terminal (BET) family, serves as a critical transcriptional coactivator that regulates gene expression. Its dysregulation is frequently observed in various cancers. ARV-825, a PROTAC composed of the CRBN ligand pomalidomide linked to the BRD4 inhibitor OTX015, has demonstrated potent BRD4 degradation capacity and significant anti-proliferative effects in MM cells (Lim et al., 2019). In MM pathogenesis, MYC oncogene dysregulation—mediated by BRD4—correlates with poor prognosis, making it an attractive therapeutic target. Another BRD4-targeting PROTAC, MZ-1 (incorporating a VHL ligand), effectively suppresses MYC levels in MM cells. Notably, MZ-1 exhibits synergistic anti-MM activity when combined with the MEK1/2 inhibitor trametinib through abrogating MYC protein levels as well as ERK2 phosphorylation levels, both *in vitro* and *in vivo* (Lind et al., 2024).

Additionally, the overexpression of pro-survival protein MCL1 has been identified as a crucial survival mechanism in MM. The first MCL1-targeting PROTAC, dMCL1-2, combines thalidomide (a CRBN ligand) with the MCL1 inhibitor A-1210477, effectively inducing MCL1 degradation in MM cells (Li and Song, 2020).

### 3.4 P53-MDM2 interaction inhibitors

#### 3.4.1 Nutlin-3

Nutlin-3, the first-in-class MDM2 inhibitor, disrupts the p53-MDM2 interaction by competitively binding to MDM2, thereby stabilizing p53 and activating the p53 signaling pathway. Studies have shown that Nutlin-3 induces apoptotic cell death in wild-type p53 (wt-p53) MM cell lines and primary patient samples. Mechanistically, this is mediated by upregulation of p53, p21, and MDM2 protein levels with a simultaneous increase in pro-apoptotic factors (PUMA, Bax, Bak), downregulation of anti-apoptotic proteins (Bcl2, survivin) and caspase cascade activation. Additionally, Nutlin-3 triggers p53 transcriptional-independent pathways, further contributing to its tumor-suppressive effects (Saha et al., 2010a). These findings highlight the therapeutic potential of nongenotoxic p53 pathway activation in MM. Importantly, Nutlin-3 exhibits synergistic activity with conventional anti-MM agents, including the proteasome inhibitor bortezomib (Ooi et al., 2009), reinforcing its clinical relevance as a promising combination therapy strategy. The addition of MDM2 inhibition to proteasome inhibition not only further stabilizes p53 and increases its protein levels, but also blocks its ubiquitination and transcriptional inactivation, thereby enhancing its functional activity.

#### 3.4.2 RITA

RITA is a small-molecule inhibitor that disrupts the p53-MDM2 interaction by directly binding to p53, leading to p53 stabilization and pathway activation. In wt-p53 MM cell lines and primary patient samples, RITA triggers apoptosis through upregulation of proapoptotic NOXA, downregulation of antiapoptotic Mcl-1, and caspases activation. These findings are further corroborated in an MM xenograft mouse model, reinforcing RITA’s therapeutic potential. Interestingly, RITA’s mechanisms extend beyond p53 reactivation. Recent studies reveal that it can also induce G2/M cell cycle arrest, upregulate p53 targets (MDM-2, PUMA, NOXA) and promote PARP cleavage even in MDM2 inhibitor-resistant cells (Jones et al., 2012). Notably, RITA exhibits strong synergistic effects with Nutlin-3, significantly enhancing growth suppression in MM cells (Saha et al., 2010b). The synergistic effect likely results from Nutlin-3’s potent inhibition of p53-MDM2 interaction coupled with RITA’s p53 reactivation and other mechanisms described above. These findings suggest that dual targeting of p53 restoration and MDM2 inhibition represent a rational and promising strategy for MM treatment.

### 3.5 Inhibitors of apoptosis proteins

Inhibitors of apoptosis proteins (IAPs) represent a family of endogenous regulators that suppress programmed cell death. Key members, including XIAP, cIAP1, cIAP2, ILP2, and livin, contain a RING finger domain that enables them to ubiquitinate and degrade caspases and the pro-apoptotic protein SMAC (second mitochondrial activator of caspases). It has been shown that XIAP is the only member of the IAP family that binds and inhibits the activation of caspases 9 and 3 (Eckelman et al., 2006). cIAP1 and cIAP2 facilitate cell survival and inhibit apoptosis by functioning as E3 ligases that promote RIP1 ubiquitination and subsequent TNF-α-induced NF-κB activation (Varfolomeev et al., 2008; Bertrand et al., 2008). In MM, elevated expression of cIAP1, cIAP2, and XIAP has been associated with poor prognosis and the development of drug resistance (Nakagawa et al., 2006). RNA interference-mediated downregulation of XIAP enhances chemosensitivity in myeloma cell lines and suppresses tumorigenesis in BPD/SCID mouse models (Desplanques et al., 2009). Smac mimetics are compounds that bind the IAPs at Smac binding sites and promote apoptosis. The SMAC mimetic birinapant demonstrates synergistic activity with bortezomib in MM cells, including bortezomib-resistant populations. This synergistic effect is mediated through cIAP1/2 downregulation rather than canonical NF-κB pathway inhibition, leading to activation of the extrinsic apoptotic pathway both *in vitro* and *in vivo* (Zhou et al., 2019). The SMAC mimetic LCL161 induces cytotoxicity and cell apoptosis in a subset of MM cell lines through downregulation of both XIAP activity and cIAP1 levels. Resistance to LCL161 is attributed to the failure of cIAP2 downregulation and pStat3 upregulation following treatment. However, combination therapy with LCL161 and a JAK2-specific inhibitor shows synergistic anti-MM effects, overcoming this resistance mechanism (Ramakrishnan et al., 2014). Currently, LCL161 is being evaluated in a phase II clinical trial for MM patients (https://clinicaltrials.gov), highlighting its translational potential. These findings underscore the therapeutic promise of SMAC mimetics as both monotherapy and combination agents in MM, warranting further clinical investigation.

## 4 Conclusions and prospects

As the core component of protein ubiquitination, E3 ubiquitin ligases are involved in the pathogenesis of MM and are therefore considered as potential therapeutic targets in MM. This review systematically summarizes the regulatory network of E3 ubiquitin ligases in MM and their therapeutic translational potential. Most of the agents targeting ubiquitin ligases are still under preclinical research, while a few are currently under clinical investigation for the treatment of MM. Collectively, these advances drive the transformation of MM treatment paradigms from traditional nonspecific chemotherapy to precision-targeted therapy.

Notwithstanding remarkable advances in E3 ubiquitin ligase-targeted therapies for MM, several critical challenges persist: (1) The emergence of CRBN mutations or downregulation-mediated resistance has become a predominant obstacle for CRBN-directed agents; (2) The limited spectrum of druggable E3 ligases; (3) PROTACs encounter substantial pharmaceutical challenges, including excessive molecular weight, limited oral absorption and unfavorable pharmacokinetic profiles; (4) Monotherapies with MDM2 inhibitors or IAPs demonstrate inadequate efficacy and narrow therapeutic indices; (5) Potential toxicities arising from off-target effects.

Consequently, great efforts should be put in the optimization and development of new molecules targeting E3 ubiquitin ligases. For examples, developing novel CELMoDs and E3 ubiquitin ligases, implementing combination therapies and advancing next-generation protein degradation technologies such as molecular glues. The E3-deubiquitinases regulatory axis functions as a molecular switch controlling protein homeostasis, with its dysregulation being pathogenic in MM (Lei et al., 2021). Combinatorial targeting via PROTACs and deubiquitinases inhibitors emerges as a promising therapeutic paradigm. Furthermore, several scientific questions demand urgent resolution, including differential sensitivity of MM subtypes to E3 ligase-targeted therapies and the development of predictive biomarkers for treatment response. In summary, as pivotal regulators in MM therapeutics, researches on E3 ubiquitin ligases have evolved from fundamental mechanistic investigations to clinical translation. With the progressive implementation of these innovative strategies, E3 ligase-targeted therapies are poised to achieve transformative breakthroughs in MM management. From a long-term perspective, deepened understanding of the E3 ligases system will not only improve clinical outcomes for MM patients but may also establish novel therapeutic paradigms for other malignancies.

## Supplementary material

SUPPLEMENTARY FIGURE S1 Genetic mutation types and frequencies of E3 ubiquitin ligases in MM based on The Cancer Genome Atlas database by using the cBioPortal tool.
