# Supplementary figures and images for: Regulatory role of E3 ubiquitin ligases in multiple myeloma: from molecular mechanisms to therapeutic strategies
Source: Front Cell Dev Biol. 2025 Jul 30;13:1620097. doi: 10.3389/fcell.2025.1620097 (PMC12343653; doi:10.3389/fcell.2025.1620097)

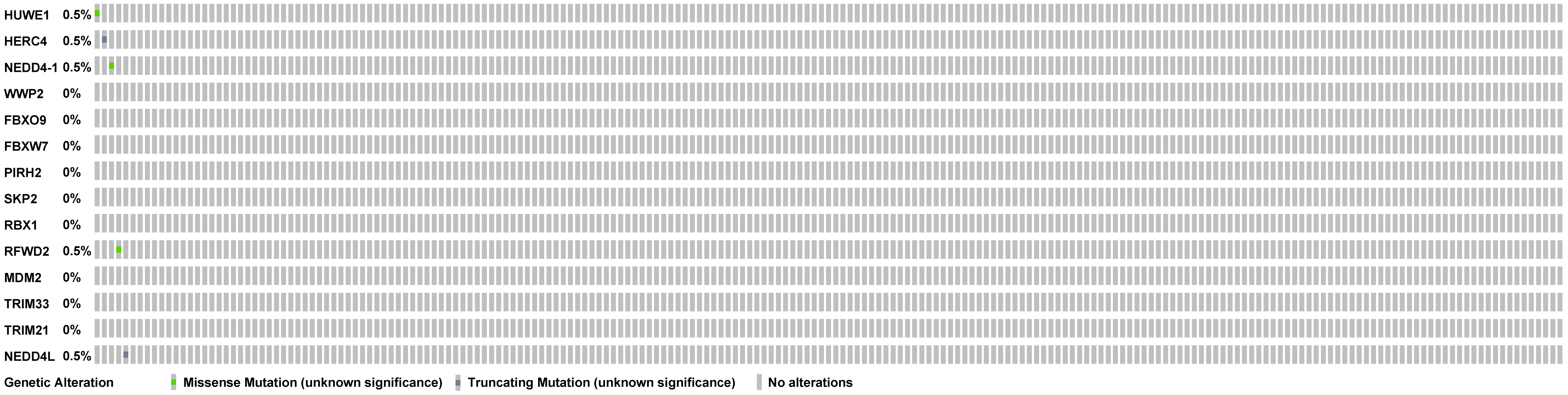

Supplement: Supplementary file 1 [file Image1.tif]
